# A super-voxel-based method for generating surrogate lung ventilation images from CT

**DOI:** 10.3389/fphys.2023.1085158

**Published:** 2023-04-26

**Authors:** Zhi Chen, Yu-Hua Huang, Feng-Ming Kong, Wai Yin Ho, Ge Ren, Jing Cai

**Affiliations:** ^1^ Department of Health Technology and Informatics, The Hong Kong Polytechnic University, Hong Kong, China; ^2^ Department of Clinical Oncology, Queen Mary Hospital, Hong Kong, China; ^3^ Department of Clinical Oncology, The University of Hong Kong, Hong Kong, China; ^4^ Department of Nuclear Medicine, Queen Mary Hospital, Hong Kong, China

**Keywords:** ventilation, 4DCT, super-voxel, radiotherapy, lung cancer

## Abstract

**Purpose:** This study aimed to develop and evaluate ${CTVI}_{SVD}$ **,** a super-voxel-based method for surrogate computed tomography ventilation imaging (CTVI).

**Methods and Materials:** The study used four-dimensional CT (4DCT) and single-photon emission computed tomography (SPECT) images and corresponding lung masks from 21 patients with lung cancer obtained from the Ventilation And Medical Pulmonary Image Registration Evaluation dataset. The lung volume of the exhale CT for each patient was segmented into hundreds of super-voxels using the Simple Linear Iterative Clustering (SLIC) method. These super-voxel segments were applied to the CT and SPECT images to calculate the mean density values (*D*_*mean*_) and mean ventilation values (*Vent*_*mean*_), respectively. The final CT-derived ventilation images were generated by interpolation from the *D*_*mean*_ values to yield ${CTVI}_{SVD}$ . For the performance evaluation, the voxel- and region-wise differences between ${CTVI}_{SVD}$ and SPECT were compared using Spearman’s correlation and the Dice similarity coefficient index. Additionally, images were generated using two deformable image registration (DIR)-based methods, ${CTVI}_{HU}$ and ${CTVI}_{Jac}$ , and compared with the SPECT images.

**Results:** The correlation between the *D*_*mean*_ and *Vent*_*mean*_ of the super-voxel was 0.59 ± 0.09, representing a moderate-to-high correlation at the super-voxel level. In the voxel-wise evaluation, the ${CTVI}_{SVD}$ method achieved a stronger average correlation (0.62 ± 0.10) with SPECT, which was significantly better than the correlations achieved with the ${CTVI}_{HU}$ (0.33 ± 0.14, *p* < 0.05) and ${CTVI}_{Jac}$ (0.23 ± 0.11, *p* < 0.05) methods. For the region-wise evaluation, the Dice similarity coefficient of the high functional region for ${CTVI}_{SVD}$ (0.63 ± 0.07) was significantly higher than the corresponding values for the ${CTVI}_{HU}$ (0.43 ± 0.08, *p* < 0.05) and ${CTVI}_{Jac}$ (0.42 ± 0.05, *p* < 0.05) methods.

**Conclusion:** The strong correlation between ${CTVI}_{SVD}$ and SPECT demonstrates the potential usefulness of this novel method of ventilation estimation for surrogate ventilation imaging.

## 1 Background

Lung cancer is the most common cause of cancer-related death in both men and women (Wild et al., 2020). Radiotherapy (RT) is an important treatment modality for lung cancer, especially in patients in whom surgical resection is contraindicated or those with mid- or late-stage lung cancers (Gadgeel et al., 2012). The functional lung volume that can be irradiated in such patients is limited, as irradiation of functioning tissue can lead to radiation pneumonitis (RP) and respiratory failure. Currently, the percentage of the lung volume receiving at least 20 Gy (*V20*) and the mean lung dose (*MLD*) are used to predict the risk of pulmonary injury (Lee et al., 2003) or the maximum acceptable dose to deliver to a lesion (Baisden et al., 2007). However, these parameters are evaluated across the whole lung volume and do not account for functional differences between lung regions. Recently, regional lung functionality assessment has been shown to enable highly functional lung areas to be spared from irradiation and thus can be used to design treatment plans that reduce the risk of injury (Hoover et al., 2014; Bucknell et al., 2018; Lee and Park, 2020; Vinogradskiy et al., 2022).

Lung ventilation images can provide regional functional information. Clinical-standard lung ventilation imaging techniques require radioactive gases or aerosols; for example, single-photon emission computed tomography (SPECT) uses Technetium-99 m (Tc-99 m) (Suga et al., 2004) and positron emission tomography (PET) uses Gallium-68 (Ga-68) (Ament et al., 2013). However, not all hospitals can perform PET or SPECT scans, and the radiopharmaceuticals used for imaging expose patients to additional radiation doses. Hyperpolarized noble gas magnetic resonance imaging (MRI) ventilation (Cai et al., 2007; Cai et al., 2009; Tustison et al., 2010; Roos et al., 2015) is another non-invasive imaging technique used to generate ionizing radiation-free ventilation images for lung function assessment. However, MRI ventilation requires a tracer gas and specialized equipment, which may limit the availability of this modality in clinical practice. CT-derived ventilation imaging (CTVI) is another method of generating ventilation images. Moreover, as CT scans of patients undergoing RT are routinely performed, CTVI methods could potentially help patients avoid unnecessary radiation doses and medical costs.

Current CTVI methods are mainly based on volume changes (Jacobian-based, ${CTVI}_{Jac}$ ) or density changes ( ${CTVI}_{HU}$ ) and use four-dimensional CT (4DCT) and deformable image registration (DIR) (Vinogradskiy, 2019). In 4DCT-based methods, the peak-inhale phase CT (CT_in_) and peak-exhale phase CT (CT_ex_) are selected from 4DCT data to represent the largest regional volume differences and changes in HU values. The rationale underlying density change-based methods is that each lung CT voxel represents a combination of water-like and air-like tissues (Simon, 2000), so the density of the lung voxel in the CT_in_ decreases when air is inhaled. The density change in each voxel then can be calculated by applying DIR to map the voxels between CT images of inhalation and exhalation. The Jacobian-based methods use the volume change in a given lung voxel due to inhaled air. The volume change can be calculated as the Jacobian of the generated DIR (Reinhardt et al., 2008). However, because these methods are performed at the voxel level, their results are substantially affected by image artifacts and DIR accuracy. Therefore, sub-regional level analysis methods have been developed to improve the accuracy robustness of CTVI (Szmul et al., 2019; Castillo et al., 2020). These methods have yielded some improvements but they also are DIR-based, which means that their accuracy depends on DIR algorithms; thus, they are affected by the parameters of DIR algorithms and the sensitivity of DIR to 4DCT image artifacts. Other CTVI methods that do not use DIR have been devised. For example, Kipritidis et al. (2016) devised a modified Hounsfield unit (HU)-based method that generates robust ventilation images without DIR. However, this method may overestimate areas with edges between solid tissue and normal parenchyma within the lung, such as the peritumoral lung and the pleural space. Some deep learning-based methods can generate highly accurate functional lung images (Zhong et al., 2019; Liu et al., 2020; Ren et al., 2021a; Ren et al., 2021b), but these results lack anatomical explanations.

Current DIR-based CTVI methods are sensitive to both CT image quality and DIR algorithms, so the images they generate have a limited correlation with the gold-standard ventilation images generated using SPECT and PET (Vinogradskiy, 2019). Consequently, the results of CTVI are complicated and difficult to interpret, meaning they may be unsuitable for clinical application. The super-pixel concept was first proposed and developed as an image segmentation technology in 2003 (Ren and Malik, 2003). It uses pixel blocks that form specific patterns with adjacent pixels that have a similar texture, color, and other features. Images can be represented by a small number of super-pixels, which significantly reduces the complexity of image post-processing. A similar concept, the super-voxel, is used for three-dimensional (3D) image analysis. An air exchange unit is evaluated using a volume of approximately 2 cm^3^ (Levin et al., 2017) that contains a cluster of CT voxels with a resolution of approximately 1 mm × 1 mm × 3 mm. The CT image of a patient with lung cancer can be pre-processed by segmentation into a small number of super-voxels, where each super-voxel contains a cluster of voxels with similar features and forms perceptually meaningful anatomic features. Drawing on this principle, the current study devised a super-voxel-based method for generating robust lung ventilation images from the mean CT density value (*D*_*mean*_) of super-voxels. The ventilation images generated are based on CT image features in the absence of DIR. The results are robust and expected to be directly interpretable and meaningful for predicting the outcomes of patients with lung cancer.

## 2 Materials and methods

### 2.1 Workflow of the study

**FIGURE 1:**
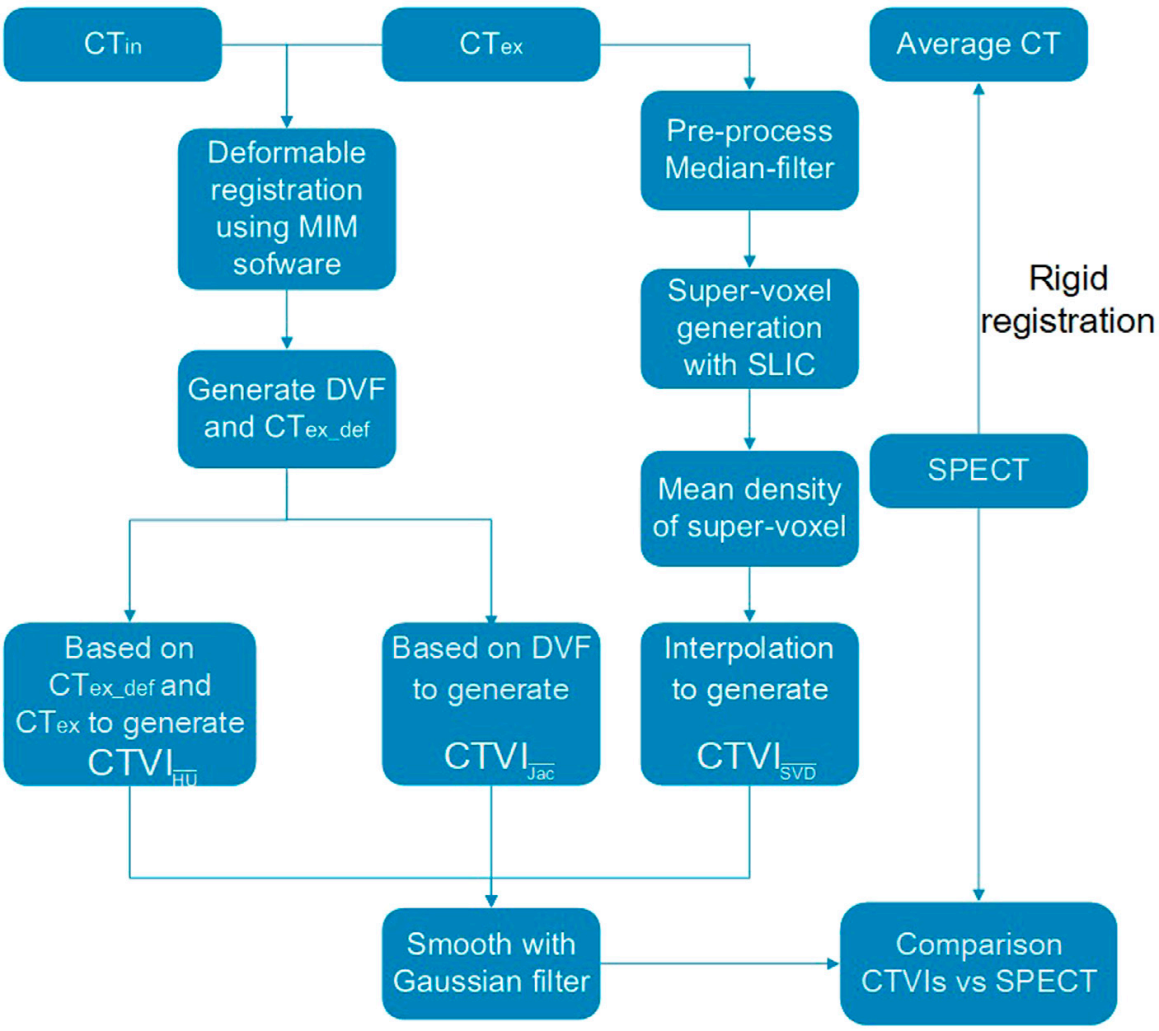
Flowchart of this study for comparing the ${CTVI}_{SVD}$ , ${CTVI}_{HU}$ , and ${CTVI}_{Jac}$ with SPECT.

Figure 1 shows the main workflow of this study. The CT_ex_ and CT_in_ were used to calculate the ventilation images. A clustering method was used to generate super-voxels, and the *D*_*mean*_ of each super-voxel was used to calculate the ventilation images ${CTVI}_{SVD}$ . The results of ${CTVI}_{SVD}$ and the DIR-based CTVIs ( ${CTVI}_{HU}$ ; ${CTVI}_{Jac}$ ) were compared with SPECT images. The details are presented in the following sections.

### 2.2 Image data

The data of 21 patients with lung cancer were acquired from the Ventilation And Medical Pulmonary Image Registration Evaluation (VAMPIRE) dataset (Kipritidis et al., 2019). All of the patients underwent 4DCT and diethylenetriamine pentaacetate (DTPA)-SPECT scans at Stanford University, United States (Yamamoto et al., 2014). All of the patients provided written informed consent to participate in a clinical trial of 4DCT ventilation imaging approved by the institutional review board for a study by Yamamoto (Yamamoto et al., 2014). Ten breathing phase CT images and a time-average CT with a slice thickness of 2.0, 2.5, or 3.0 mm were available for each patient. The average interval between the 4DCT and subsequent DTPA-SPECT (including low-dose attenuation correction CT) scans was 4 (±5) days. Rigid registration was performed between each SPECT image and the time-average CT image using Mattes mutual information rigid registration in Plastimatch. The DTPA-SPECT scans were linearly interpolated to match the dimensions of the time-average CT image (Kipritidis et al., 2019). The lung masks for all of the CT images (4DCT and attenuation correction CT) were also acquired from the VAMPIRE dataset, which used a region-growing method. The lung masks of the attenuation correction CT images were also used as the masks of the SPECT images. The CT values were converted to density values using Eq. 1, as follows:

$$Density=\frac{HU+1000}{1000}$$ (1)

### 2.3 DIR-based CTVI methods

The two main conventional DIR-based methods are ${CTVI}_{HU}$ and ${CTVI}_{Jac}$ . Both methods require DIR between the CT_in_ and CT_ex_. In ${CTVI}_{HU}$ , a voxel at spatial position x of the CT_ex_ is mapped toward a voxel at spatial position $x^{\prime }$ of the CT_in_ by DIR. The ventilation value at position x can be directly calculated using Eq. 2 (Kipritidis et al., 2019), as follows:

$$Vent\left(x\right)=\frac{-1000\times \left({HU}_{ex}\left(x\right)-{HU}_{in}\left(x^{\prime }\right)\right)}{{HU}_{ex}\left(x\right)\times \left({HU}_{in}\left(x^{\prime }\right)+1000\right)}$$ (2)

In ${CTVI}_{Jac}$ , the volume change of a voxel at position x is calculated using the determinant of the Jacobian of the deformation field at position x . This process is performed using Eq. 3, as follows:

$$Vent\left(x\right)=\left|\begin{array}{ccc} 1+\frac{\partial u_{x}\left(x\right)}{\partial x} & \frac{\partial u_{x}\left(x\right)}{\partial y} & \frac{\partial u_{x}\left(x\right)}{\partial z}\\ \frac{\partial u_{y}\left(x\right)}{\partial x} & 1+\frac{\partial u_{y}\left(x\right)}{\partial y} & \frac{\partial u_{y}\left(x\right)}{\partial z}\\ \frac{\partial u_{z}\left(x\right)}{\partial x} & \frac{\partial u_{z}\left(x\right)}{\partial y} & 1+\frac{\partial u_{z}\left(x\right)}{\partial z} \end{array}\right|-1$$ (3)

Both ${CTVI}_{HU}$ and ${CTVI}_{Jac}$ images were calculated in this study and used for comparison. DIR between the CT_in_ and CT_ex_ was performed using MIMvista 6.3.4 (MIM Software Inc., Cleveland, OH, United States) with a default spacing resolution of 3 mm.

### 2.4 Super-voxel segmentation

Simple linear iterative clustering (SLIC) (Achanta et al., 2012) is a clustering method applied to lung CT 3D images to generate super-voxels with low computational power requirements. The SLIC algorithm first initializes the $K_{init}$ seeds by resampling pixels on a regular grid. Then, it assigns each voxel to the closest seed point to generate $K_{init}$ clusters based on the distance (D), as described by Eq. 4:

$$D=\sqrt{{d_{c}}^{2}+{\left(\frac{d_{s}}{S}\right)}^{2}\times m^{2}}$$ (4)

where $d_{c}$ is the HU value difference, $d_{s}$ is the Euclidean distance, *S* is the initial sampling interval $S=\sqrt{\frac{N}{K_{init}}}$ , *N* is the total voxel number in the lung volume, and m is a weighting value used to control the compactness of the super-voxel. Next, the positions of the centers are moved to the point with the smallest gradient to prevent placement on the edges of an image or at a noisy voxel. The above steps are repeated until the result converges. Only the super-voxels in the lung mask were used in this study. An in-house tool based on Matlab (MathWorks Inc., Natick, MA, United States) was used, and $K_{init}$ was set as 1,500 for all of the patients (refer to the Discussion section for commentary). The number of super-voxels generated varied between the patients according to their lung anatomy. All of the CT and SPECT images were interpolated into images of the same size and with a pixel size of with 2 mm ✕ 2 mm ✕ 2 mm, and a 3D median filter with dimensions of 5 voxels ✕ 5 voxels ✕ 5 voxels was applied to the images to reduce noise.

### 2.5 Super-voxel-based ventilation image ${CTVI}_{SVD}$ calculation

**FIGURE 2:**
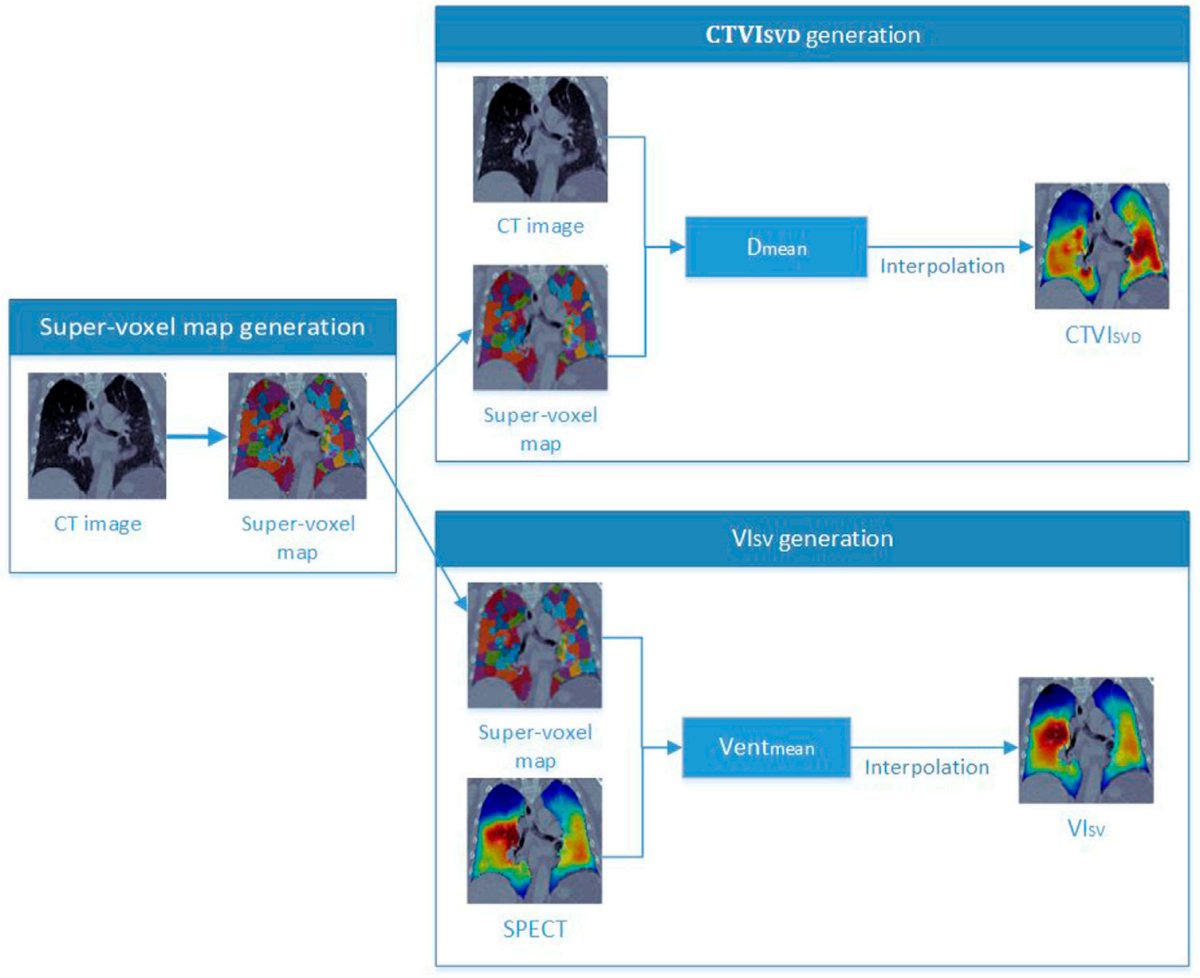
The workflow of the generations of the ${CTVI}_{SVD}$ and ${VI}_{SV}$ .

As shown in Figure 2, a super-voxel map was generated on CT_ex_ images (as described in Section 2.4), and the *D*_*mean*_ of each super-voxel was calculated. Other studies have used fixed threshold intervals of −1,024 to −400 HU to generate the lung parenchyma (Kemerink et al., 1998; Kuhnigk et al., 2005). In the current study, the same fixed threshold interval was applied to identify the non-lung region; a super-voxel with a *D*_*mean*_ greater than 0.6 according to Eq. 1 was assigned a value of 0 to remove clearly false results from consolidation of the tumor and abnormal tissues, which have a high density but should have a low ventilation value. The super-voxel segmentation results were then directly mapped on the SPECT images, as both the SPECT and time-average CT data were registered according to the VAMPIRE challenge, and the time-average CT and CT_ex_ images shared the same position. The mean ventilation value (*Vent*_*mean*_) was calculated using SPECT image data. The correlation between the *D*_*mean*_ and *Vent*_*mean*_ of the super-voxels was determined using Spearman’s correlation analysis.

Figure 2 shows the workflow for generating the ${CTVI}_{SVD}$ images and the ventilation images based on SPECT ( ${VI}_{SV}$ ). ${CTVI}_{SVD}$ image generation requires only a CT_ex_ image, while ${VI}_{SV}$ images require both CT_ex_ and SPECT images. To demonstrate the feasibility of generating a reasonable ventilation image using hundreds of super-voxels, we generated the VI_SV_ image and compared it with a SPECT image. The details of ${CTVI}_{SVD}$ are presented as follows. To perform ${CTVI}_{SVD}$ of the whole lung volume, we used the geometric center of a super-voxel to represent the position of the super-voxel, and the *D*_*mean*_ value as the ventilation value of the center positions of the super-voxels. The ventilation values of all of the voxels in a lung were then calculated via interpolation with the *D*_*mean*_ of the super-voxels, as follows (Eq. 5):

$$V=WV_{\sup}$$ (5)

$$w_{ij}=e^{-{\left(\frac{r_{ij}}{r_{mean}}\right)}^{2}}$$ (6)

where V is the vector of the ventilation value of all voxels in a lungs; $V_{\sup}$ is the vector calculated only using the *D*_*mean*_ of the super-voxel; W is the interpolation weight matrix; $w_{ij}$ is the element of the W matrix, which is calculated based on the distance between voxel i and the center position of super-voxel j , as shown in Eq. 6; $r_{mean}$ is the mean distance between the super-voxels; and $r_{ij}$ is the distance between voxel i and super-voxel j . The lung volume was divided into the left and right lungs. For each voxel, the ventilation value was interpolated using only the super-voxels from the ipsilateral lung. To smooth the final CTVIs, we applied a 3D Gaussian filter with a kernel size of three voxels to each lung voxel. The same post-processing steps were applied to all CTVIs. The *Vent*_*mean*_ of the super-voxels from SPECT was used to generate the ${VI}_{SV}$ according to the above-stated interpolation method and the correlation between ${VI}_{SV}$ and SPECT was evaluated. Two more super-voxels-based ventilation images were also generated for comparison. The *Vent*_*mean*_ of the super-voxels from ${CTVI}_{HU}$ and ${CTVI}_{Jac}$ was used to generate the ${CTVI}_{SVHU}$ and ${CTVI}_{SVJac}$ with a similar method as ${VI}_{SV}$ , respectively. Their correlations with SPECT were also evaluated.

### 2.6 Comparison of ${CTVI}_{SVD}$ , ${CTVI}_{HU}$ , ${CTVI}_{Jac}$ , ${CTVI}_{SVHU}$ , and ${CTVI}_{SVJac}$ with SPECT

The ${CTVI}_{SVD}$ images generated in this study were evaluated with the corresponding SPECT images using voxel-wise Spearman correlation analysis. Spearman correlation analysis was also used to compare SPECT images with CT_ex_, ${CTVI}_{HU}$ , ${CTVI}_{Jac}$ , ${CTVI}_{SVHU}$ , and ${CTVI}_{SVJac}$ images. The comparison between the CT_ex_ and SPECT was used to show the advantages of analysis at the super-voxel level compared to the voxel level. To assess the concordance of high-functioning regions between CTVI and SPECT, SPECT and ${CTVI}_{SVD}$ images from each patient were divided into two volumes by the 66th percentile ventilation value in the lung, which is used to distinguish high- and low-functioning lung regions. This value has been used by other studies (Yamamoto et al., 2011; Ren et al., 2021b). The Dice similarity coefficient index (*DSC*) was used to assess the accuracy of ${CTVI}_{SVD}$ in segmenting the high- and low-functioning lung regions. The *DSC* was also used to compare the high- and low-functioning lung regions segmented by SPECT with those segmented by ${CTVI}_{HU}$ , ${CTVI}_{Jac}$ , ${CTVI}_{SVHU}$ , and ${CTVI}_{SVJac}$ . Only the intersection between the CT and SPECT lung masks was analyzed in this study.

### 2.7 Impact of the super-voxel number on ${CTVI}_{SVD}$ experiments

The size of the super-voxels may influence the results of ${CTVI}_{SVD}$ . On the one hand, super-voxels that are too large may not be able to identify small defects. On the other hand, super-voxels that are too small may lose their structure-oriented properties. A particular clustering may influence the results of ${CTVI}_{SVD}$ . For example, by increasing the number of super-voxels, the size of clusters is reduced. To investigate how the size of the super-voxels influences the results, we measured the correlation of ${CTVI}_{SVD}$ with SPECT for different numbers of super-voxels. Performance was evaluated at various values of $K_{init}$ (300, 500, 800, 1,000, 1,500, 2,000, 2,500, 3,000, 4,000, 8,000, 12,000, and 15,000) to cover an extensive range. A large value of $K_{init}$ increases the calculation time and depletes the memory needed to calculate the interpolation matrix *W*, as described in Section 2.5. The computer used for this analysis was equipped with an Intel^®^ Core™ i9-11900K 3.50-GHz processor and 64.0 GB of RAM.

## 3 Results

### 3.1 Super-voxel segmentation

**FIGURE 3:**
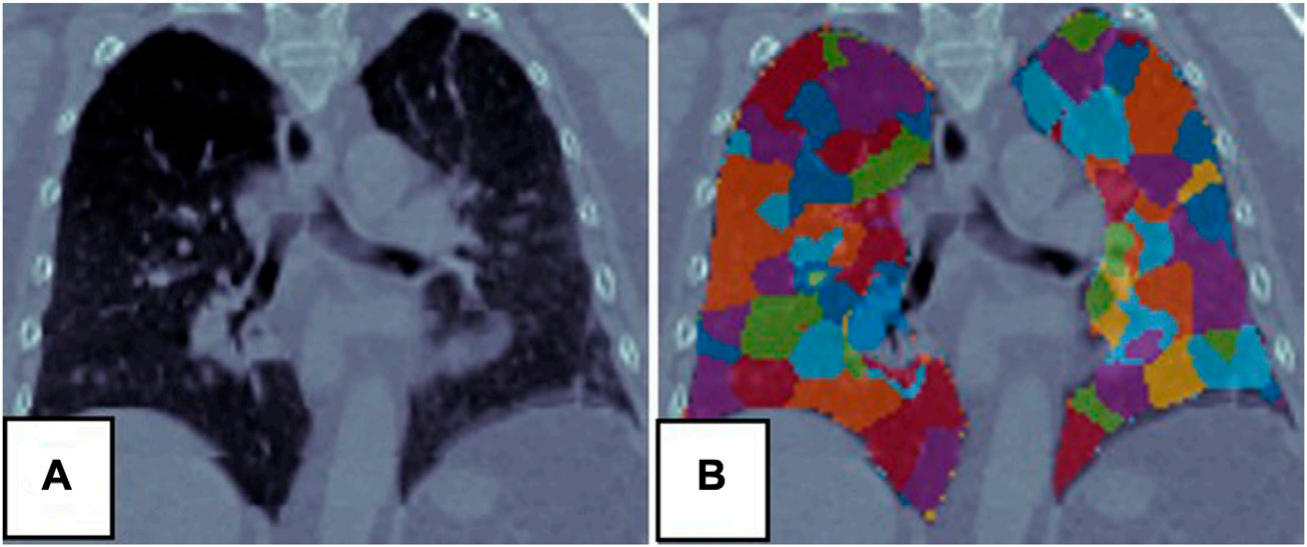
Super-voxel segmentation in the lungs of a patient. **(A)** Is the CT, **(B)** is the result of the super-voxel segmentation in the lung region.

**FIGURE 4:**
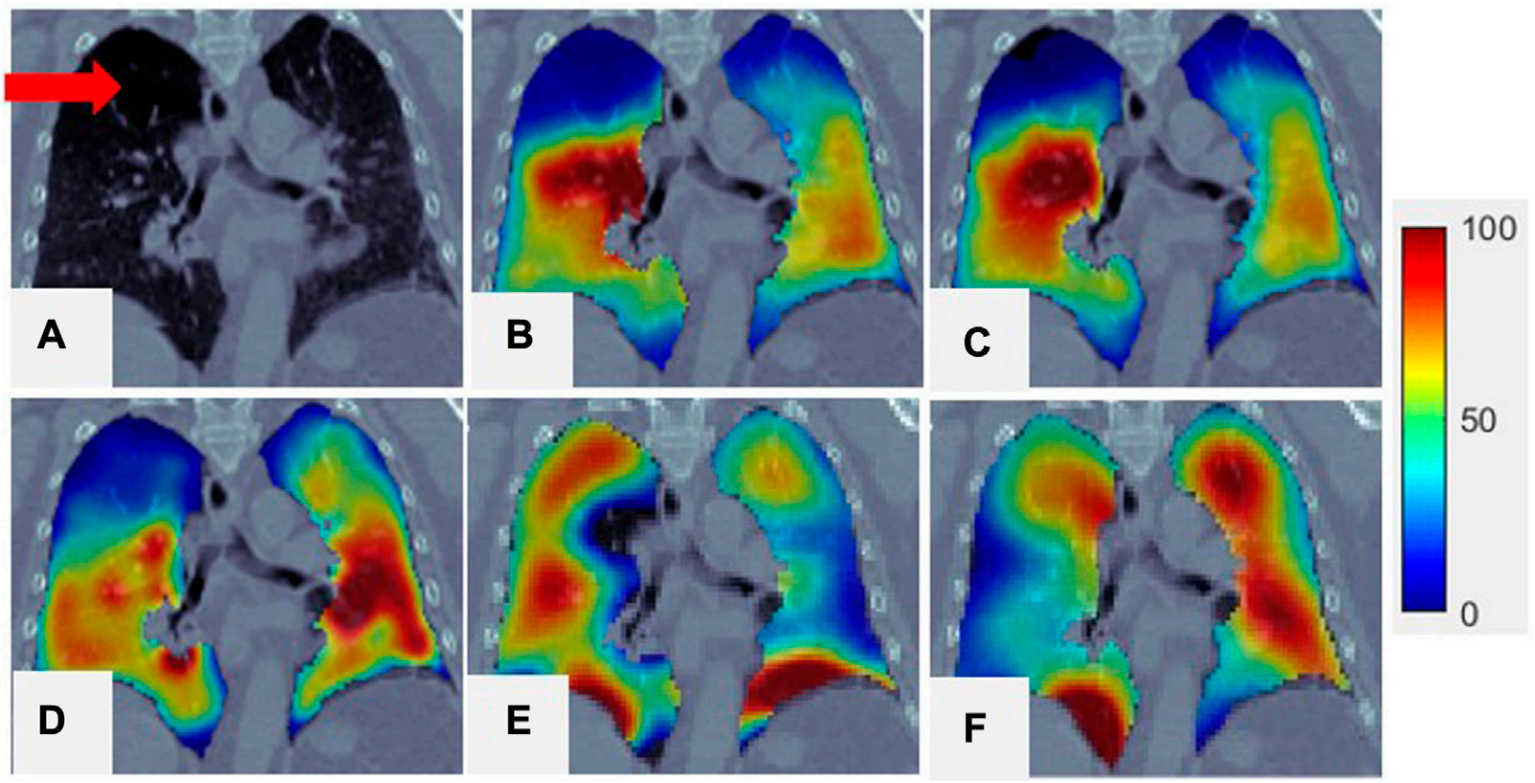
Comparison of SPECT image and ${CTVI}_{SVD}$ images for a representative case. **(A)** Is CT; **(B)** is the SPECT of the lung region superimposed onto the CT; **(C)** is the ${VI}_{SV}$ of the lung region superimposed onto the CT; **(D)** is the ${CTVI}_{SVD}$ of the lung region superimposed onto the CT; **(E)** is the ${CTVI}_{Jac}$ of the lung region superimposed onto the CT; **(F)** is the ${CTVI}_{HU}$ of the lung region superimposed onto the CT. For all the figures, their 99th percentile and higher values were scaled to 100 (to reduce the artifact effect caused by the tracer deposited at airways in SPECT for visual inspection), and the minimum value was scaled to 0.

The SLIC method was used to divide the lung volumes of the 21 patients into 380–715 super-voxels at a $K_{init}$ of 1,500. Figure 3 shows an example of super-voxel segmentation of the lung volume. Different colors indicate different super-voxel regions. The mean correlation between ${VI}_{SV}$ and SPECT was 0.91 (range: 0.84–0.96). Figures 4B, C show a comparison between SPECT and ${VI}_{SV}$ images. The two images have a similar function distribution. The strong correlation between ${VI}_{SV}$ and SPECT suggests that a reasonable CTVI image of the whole lung volume can be generated by analyzing hundreds of super-voxels.

### 3.2 Comparison of ${CTVI}_{SVD}$ , ${CTVI}_{HU}$ , ${CTVI}_{Jac}$ , ${CTVI}_{SVHU}$ , and ${CTVI}_{SVJac}$ with SPECT

The correlation between the *D*_*mean*_ from CT and the *Vent*_*mean*_ for the super-voxel volume from SPECT was 0.59 ± 0.09, indicating that super-voxels with a lower mean density tend to have a lower function value than super-voxels with a higher mean density. This moderate-to-strong correlation means that the *D*_*mean*_ of a super-voxel can be used as a surrogate for *Vent*_*mean*_ when generating ${CTVI}_{SVD}$ , as mentioned in Section 2.5. Figure 4 presents a comparison of SPECT with ${CTVI}_{SVD}$ . The low-functioning lung region, indicated by the red arrow in the CT image and by the blue and black-blue area in the ventilation image (Figure 4B), can be identified using ${CTVI}_{SVD}$ (dark blue area in Figure 4D). The mean correlation coefficient between ${CTVI}_{SVD}$ and SPECT was 0.62 (range: 0.37–0.77). The mean correlation coefficients of SPECT with CT_ex_, ${CTVI}_{HU}$ ; ${CTVI}_{Jac}$ ; ${CTVI}_{SVHU}$ ,; ${CTVI}_{SVJac}$ were 0.16 ± 0.16, 0.33 ± 0.14, 0.23 ± 0.10, 0.39 ± 0.18, and 0.33 ± 0.15 respectively. These results indicate that ${CTVI}_{SVD}$ is closer to SPECT than conventional DIR-based methods. The super-voxel based method can improve the correlations of the DIR-based CTVIs by 0.06 and 0.10 for ${CTVI}_{HU}$ ; ${CTVI}_{Jac}$ , respectively. A similar improvement was also reported by Szmul’s study (Szmul et al., 2019).

The mean *DSC* values of the high-functioning ( ${DSC}_{h}$ ) and low-functioning regions ( ${DSC}_{l}$ ) on ${CTVI}_{SVD}$ images were 0.63 ± 0.07 and 0.81 ± 0.03, respectively. Because the criterion for dividing the lung is the 66th, the low-functioning region is larger than the high-functioning region, and ${DSC}_{l}$ is higher than ${DSC}_{h}$ . As shown in Figure 4, the locations of the low-functioning regions on the ${CTVI}_{SVD}$ images matched those on the SPECT images, but the highest-functioning regions (dark red area) just exhibited a certain amount of overlap. The mean ${DSC}_{h}$ values of ${CTVI}_{HU}$ , ${CTVI}_{Jac}$ , ${CTVI}_{SVHU}$ , and ${CTVI}_{SVJac}$ were 0.43 ± 0.08 and 0.42 ± 0.05, 0.49 ± 0.11, and 0.48 ± 0.07, respectively, and the corresponding mean ${DSC}_{l}$ values were 0.70 ± 0.04, 0.70 ± 0.03, 0.74 ± 0.06, and 0.73 ± 0.04, respectively.

**FIGURE 5:**
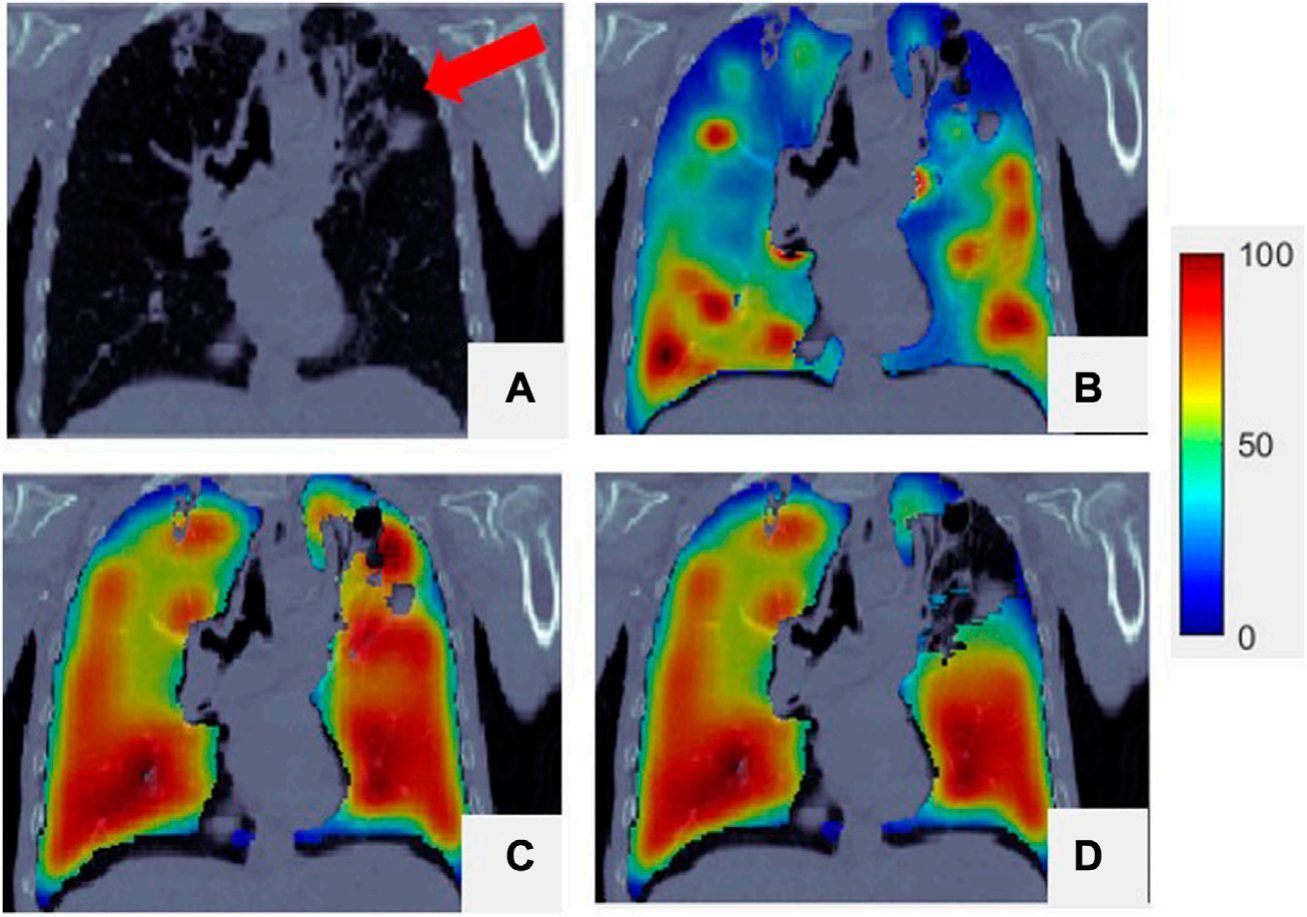
Comparison of SPECT image and ${CTVI}_{SVD}$ images for a representative case. **(A)** Is CT; **(B)** is the SPECT of the lung region superimposed onto the CT; **(C)** is the origin ${CTVI}_{SVD}$ of the lung region superimposed onto the CT; **(D)** is the corrected ${CTVI}_{SVD}$ of the lung region superimposed onto the CT. For all the figures, their 99th percentile and higher values were scaled to 100, and the minimum value was scaled to 0.

For some patients, ${CTVI}_{SVD}$ yielded low correlation with SPECT. However, this could be improved. As indicated by the red arrow in Figure 5A, a defective lung region with a high density at the top of the left lung caused a falsely high ventilation value, as shown in Figure 5C. Such errors can be corrected by manually contouring the defect regions via assignment to a low ventilation value. In this case, the final correlation coefficient increased to 0.52, as shown in Figure 5D.

### 3.3 Evaluation of the impact of the super-voxel number on ${CTVI}_{SVD}$

**FIGURE 6:**
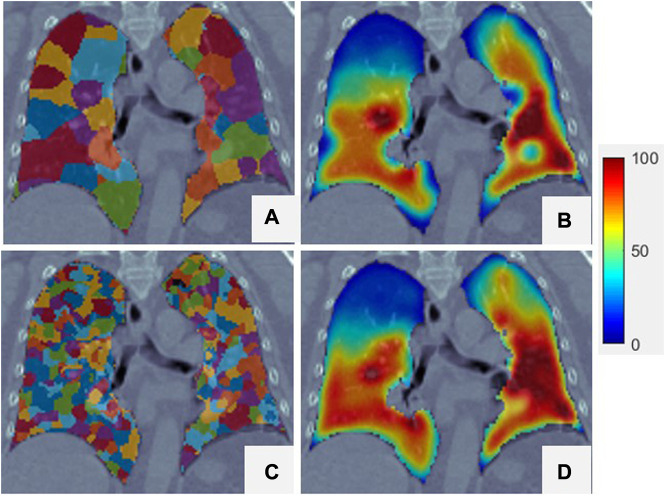
Two different super-voxel segmentations with different $K_{init}$ and the corresponding ${CTVI}_{SVD}$ . The $K_{init}$ of the top row is 500, and the bottom row is 120000. **(A,C)** are the results of the super-voxel segmentation in the lung region. **(B,D)** are the CTVI_SVD_ of the lung region superimposed onto the CT.

Figure 6 shows super-voxel segmentation using two values of $K_{init}$ . As the number of super-voxels increased, the size of the super-voxels decreased. The generated ${CTVI}_{SVD}$ images show high similarity in highly ventilated regions. As shown in the bottom left row of Figure 6, as the volume of the super-voxel decreased, it became more difficult to contain the whole texture of the sub-region; this presents an obstacle to analysis of the *Vent*_*mean*_ with other features of such a super-voxel. Table 1 shows the experimental results obtained with different numbers of super-voxels. On average, approximately 193, 280, 373, 413, 520, 615, 713, 802, 1,018, 1,788, 2,550, and 3,108 super-voxels were extracted from the lung volumes of the 21 patients when $K_{init}$ was set as 300, 500, 800, 1,000, 1,500, 2,000, 2,500, 3,000, 4,000, 8,000, 12,000, and 15,000, respectively. The correlation of *D*_*mean*_ with *Vent*_*mean*_ was strongest when approximately 520 super-voxels were extracted from the lung volume and decreased as the number of super-voxels continued to increase. A paired-samples *t*-test to compare the *D*_*mean*_ and *Vent*_*mea*n_ obtained at a $K_{init}$ of 1,500 with those obtained at other $K_{init}$ values revealed that a $K_{init}$ of 1,500 generated the most reasonable number of super-voxels inside the lungs. The *D*_*mean*_ exhibited a stronger correlation with *Vent*_*mean*_ at a $K_{init}$ of 1,500 than at $K_{init}$ values lower than 1,500 and higher than 3,000. The correlation of ${CTVI}_{SVD}$ with SPECT reached a plateau at approximately 520 super-voxels and remained stable as the number of super-voxels increased. Thus, $K_{init}$ was set as 1,500 to retain as many structure-oriented properties as possible for each super-voxel.

**TABLE 1:** The influence of the different numbers of the super-voxel. $K_{init}$ means the initial setting of the super-voxel number for the CT image, and $K_{final}$ means the final extracted super-voxel number in the lung volume. The mean correlation value is the mean Spearman correlation value of all the patients. *D*_*mean*_ is the mean density of the super-voxel, and *Vent*_*mean*_ is the mean ventilation value of the super-voxel. The *p*-values are obtained from the paired-samples *t*-test of the $K_{init}$ of other value with the $K_{init}$ of 1,500.

$K_{init}$	$K_{final}$	Mean correlation value			
		*D*_*mean*_ vs. *Vent*_*mean*_	*p*-value	${CTVI}_{SVD}$ vs. SPECT	*p*-value
300	193	0.49	0.0001	0.57	0.0002
500	280	0.50	0.0002	0.58	0.0003
800	373	0.57	0.0146	0.61	0.0915
1,000	413	0.57	0.0207	0.60	0.0012
1,500	520	0.59	—	0.62	—
2,000	615	0.57	0.0739	0.62	0.2733
2,500	713	0.58	0.2508	0.63	0.0606
3,000	802	0.57	0.0948	0.63	0.0738
4,000	1,018	0.54	0.0120	0.63	0.1480
8,000	1,788	0.48	0.0005	0.62	0.3097
12,000	2,550	0.46	0.0004	0.62	0.3142
15,000	3,108	0.45	0.0001	0.62	0.3376

## 4 Discussion

In this study, a super-voxel-based method was developed to generate surrogate ventilation images directly from CT images. The SLIC method was employed to generate super-voxels inside the lung volume, and the *D*_*mean*_ of the super-voxels was used as a surrogate for the mean ventilation value to calculate a whole-lung ventilation image through interpolation. This novel ${CTVI}_{SVD}$ method achieved a mean Spearman’s correlation coefficient of 0.62 (range: 0.37–0.77) with the ground-truth SPECT, which was significantly higher than the correlation coefficients of SPECT with the DIR-based methods ${CTVI}_{HU}$ (0.33 ± 0.14, *p* < 0.05), ${CTVI}_{Jac}$ (0.23 ± 0.10, *p* < 0.05), ${CTVI}_{SVHU}$ (0.39 ± 0.18, *p* < 0.05), and ${CTVI}_{SVJac}$ (0.33 ± 0.15, *p* < 0.05). The ${DSC}_{h}$ of ${CTVI}_{SVD}$ was 0.63 ± 0.07, which was also significantly higher than those of ${CTVI}_{HU}$ (0.43 ± 0.08, *p* < 0.05), ${CTVI}_{Jac}$ (0.42 ± 0.05, *p* < 0.05), ${CTVI}_{SVHU}$ (0.49 ± 0.11, *p* < 0.05) and ${CTVI}_{SVJac}$ (0.48 ± 0.07, *p* < 0.05), and the ${DSC}_{l}$ of ${CTVI}_{SVD}$ (0.81 ± 0.03) was higher than those of ${CTVI}_{HU}$ (0.70 ± 0.04, *p* < 0.05), ${CTVI}_{Jac}$ (0.70 ± 0.03, *p* < 0.05), ${CTVI}_{SVHU}$ (0.74 ± 0.06, *p* < 0.05) and ${CTVI}_{SVJac}$ (0.73 ± 0.04, *p* < 0.05). By using this novel method, the complexity of a ventilation imaging problem can be reduced from calculating millions of ventilation values for all voxels to only calculating hundreds of *Vent*_*mean*_ values for super-voxels. The *Vent*_*mean*_ of a super-voxel can be directly derived from super-voxel features. Thus, ${CTVI}_{SVD}$ can be generated without DIR, so the novel method is simpler and more robust than DIR-based methods.

This study shows that the *D*_*mean*_ of a super-voxel is strongly correlated with the *Vent*_*mean*_ of a super-voxel, which means that a lower super-voxel density is usually associated with less functional ventilation than a higher super-voxel density. Similar results have been shown in other studies (Lafata et al., 2019; Yang et al., 2021). As shown in Figure 4, the region with low ventilation function (indicated by arrows) is darker than the region with normal function. The low-functioning region may correspond to a defective lung region caused by emphysema, where healthy pulmonary tissue has been replaced increasingly by air due to alveolar damage and weakening and rupture of the inner walls of the air sacs. This was a preliminary study of the use of the *D*_*mean*_ of super-voxels to generate ventilation images, and only 21 patients were included. Other super-voxel features can be analyzed and combined with Dmean to build a more accurate and robust model for future CTVI studies involving more patient data.

According to Eq. 4, the total super-voxel number and compactness value affect segmentation of the super-voxels. The SLIC algorithm used in this study can refine the compactness value adaptively to reduce the influence of this variable without requiring pre-assignment. The only variable required for SLIC is the total number of super-voxels. The correlation between *D*_*mean*_ and *Vent*_*mean*_ is strongest when approximately 520 super-voxels are extracted from the lung volume and decreases as the number of super-voxels increases. Meanwhile, as the number of super-voxel increases, the mean correlation between ${CTVI}_{SVD}$ and SPECT increases and then plateaus. A reasonable explanation for this observation is that as the number of super-voxels increases, the size of the super-voxels decreases, and some of the densities of these small super-voxels are then affected by the bronchi, noise, or artifacts with high-density values. The mean correlation between the *D*_*mean*_ and *Vent*_*mean*_ of the super-voxels decreases to tend to be the pixel level results, which had the same value, 0.40 ± 0.19, as the correlation between the CT_ex_ (after interpolation and denoising with a median filter) and SPECT. However, these discrepancies can be reduced by the smoothness of the Gaussian filter in the final image processing. The correlation between ${CTVI}_{SVD}$ and SPECT remains stable. Accordingly, in this study, the $K_{init}$ setting that yielded the strongest correlation between the *D*_*mean*_ and *Vent*_*mean*_ was selected to maintain the structure-oriented properties of the super-voxels to the greatest extent possible. The correlation between the CT_ex_ and SPECT was 0.16 ± 0.16, significantly lower than the result obtained with ${CTVI}_{SVD}$ method. This outcome is probably mainly attributable to the technical limitations associated with SPECT imaging. SPECT has an original resolution of 8 mm. We resampled the SPECT image with a resolution of 2 mm in the data process, so it could serve as a data smoothing process. This process was similar to our method, wherein we calculated the mean density of the super-voxel and then used interpolation to calculate the value of each voxel.

This study has some limitations. Pulmonary ventilation refers to the air exchange between the atmosphere and the lungs. It involves the inflow of air through the airway to the alveoli, where the air exchange occurs, followed by outflow through the airway. Our results show that lung regions with lower density values exhibit lower ventilation values than those with higher density values. As previously mentioned, the damaged alveoli in a patient with emphysema lost their ability to expel air, leading to decreased intensity. However, in some cases, abnormal lung regions associated with pulmonary diseases can exhibit increased density, known as opacities, and fall into four patterns: consolidation, interstitial, nodules or masses, and atelectasis (L ung-disease, 2023). These diseases can also obstruct the airway or damage to the parenchyma, leading to a loss of air exchange capability. Consequently, some pulmonary diseases may affect the CTVI results in this study. However, the clinical presentation of pulmonary diseases on CT images can vary. Raju et al. categorized the signs of the lung disease into 22 groups (Raju et al., 2017). These signs can increase the difficulty of automatically recognizing defect regions. In this study, the super-voxel was the smallest unit of analysis and its features can be used directly to classify it as a defect or normal region. In future work, we will create a super-voxel-based model to automatically identify defect regions and correct the ventilation value to increase the accuracy of our method.

Moreover, some regions may have a low ventilation value due to pressure placed by the tumor on the central airway and blood vessels; this pressure can be recovered after radiotherapy (Yuan et al., 2012). Such regions need to be carefully protected during the treatment, and the dose should be as low as possible as normal lung regions. In cases with such regions, the patient’s dyspnea may be reduced and the lung function may increase if the tumor shrinks after treatment. From this perspective, CTVI can provide more information than SPECT. More investigation is needed to identify these regions and thus guide treatment planning.

## 5 Conclusion

In this study, we developed a super-voxel-based method to generate surrogate ventilation images from CT data. The observed correlation between ${CTVI}_{SVD}$ and SPECT indicates that ${CTVI}_{SVD}$ has high similarity with SPECT. Our results also show that the *D*_*mean*_ can be used as a surrogate for the *Vent*_*mean*_ in the context of generating ventilation images.

## Data Availability

The original contributions presented in the study are included in the article/supplementary material, further inquiries can be directed to the corresponding authors.
